# Association between frailty and the combination of physical activity level and sedentary behavior in older adults

**DOI:** 10.1186/s12889-019-7062-0

**Published:** 2019-06-07

**Authors:** Venicius Dantas da Silva, Sheilla Tribess, Joilson Meneguci, Jeffer Eidi Sasaki, Cíntia Aparecida Garcia-Meneguci, José Ailton Oliveira Carneiro, Jair Sindra Virtuoso

**Affiliations:** 1 Center for Research in Physical Activity & Health, Federal University of Triangulo Mineiro, Uberaba, MG Brazil; 2 Department of Health, Southwestern State University of Bahia, Jequié, BA Brazil

**Keywords:** Frailty, Sedentary behavior, Physical activity, Older adults

## Abstract

**Background:**

The combined association of physical activity and sedentary behavior with adverse health factors is not yet clear in the literature. A combined analysis of physical activity level and sedentary behavior may provide evidence of the interrelation between these behavioral variables and the frailty syndrome. Thus, the aim of this study was to examine the relationship between physical activity level, sedentary behavior and frailty in older adults.

**Methods:**

In this study, we evaluated 457 older adults (age range = 60 to 96 years old) from the Longitudinal Study of the Elderly Health of Alcobaça, Bahia. The frailty condition was defined by the presence of three or more of the following criteria: unintentional weight loss, slow walking speed measured over a 4.57 m test, a reduction of manual grip strength and exhaustion. Based upon these criteria, participants were classified as non-frail or frail. Physical activity level and time spent in sedentary behavior were assessed with the International Questionnaire of Physical Activity. Descriptive statistics were used to characterize the sample. To examine the combined association of physical activity and sedentary behavior with frailty, chi-square and Poisson regression tests were used. Statistical significance was defined as *p* ≤ 0.05.

**Results:**

The prevalence of frailty was 8.8% (*n* = 40), with higher prevalence observed with increasing age. Low physical activity level combined with excessive time spent in sedentary behavior (physical activity level < 150 min/wk. and sedentary behavior ≥540 min/day) was associated with frailty, resulting in a prevalence ratio of 2.83 (95% CI, 1.23 to 6.52).

**Conclusion:**

Frailty is more prevalent among older adults who exhibit insufficient levels of physical activity combined with a great amount of time spent in sedentary behavior, even when adjusted for sociodemographic factors.

## Background

Frailty is a complex concept involving a state of greater vulnerability to adverse health factors, including falls, fractures, disability and an overall negative state of health [1–3], which are related to increased chances for morbidity and mortality [4]. Reducing or eliminating risk factors and increasing protective factors are potential actions for minimizing the chances of frailty [5]. Age, sex, diseases, social factors, economic factors, malnutrition, low levels of physical activity and greater time spent in sedentary behavior are known risk factors for frailty [6, 7].

Regular physical activity promotes improvements in both physical and psychological health and contributes to the reversal of detrimental effects of chronic diseases as well as the maintenance of functional autonomy in older adults [8, 9]. However, physical activity levels have been decreasing over time as a result of the increasing use of technology in society, and this decrease is considered a worldwide pandemic [10, 11]. This fact is of major concern in older adults, as they more often present with insufficient activity levels when compared to other age groups [12]. Insufficient physical activity is related to increased vulnerability to adverse health outcomes and, consequently, to higher probability for frailty in older adults [13, 14].

Research on the effect of behavior on frailty has primarily involved physical activities of moderate to vigorous intensity [15, 16]. However, the relationship between frailty and time spent in sedentary behavior in older individuals still warrants further investigation, as there is evidence of excessive sedentary behavior increasing the odds for disability [17], inflammatory processes [18] and mortality in older adults [19, 20], even in individuals who meet the recommended levels of physical activity [21, 22].

Previous studies have explored the relationship between frailty and either physical activity level or sedentary behavior alone in older adults [7, 23]. However, there is a lack of studies, in the literature that examined the association between frailty and the combination of physical activity level and sedentary behavior in older adults [6]. Understanding how these imminent risk factors at in combination in the frailty syndrome may enable the proposal of more assertive actions in promoting the maintenance of functional health in older adults. Preventing and delaying the onset of the frailty syndrome is essential for greater physical independence late in life, which is an important aspect for higher survival rates and quality of life. Therefore, the purpose of this study was to analyze the relationship between frailty syndrome and the combination of physical activity level and sedentary behavior in older adults.

## Methods

### Study design and study population

This was a cross-sectional study conducted in Alcobaça, Bahia, Brazil, as part of the project “Longitudinal Study of the Elderly Health of Alcobaça” (ELSIA), which aims to examine the life and living conditions of the older adults living in the city of Alcobaça, Bahia, Brazil.

The study population comprised 743 people of both sexes aged 60 years or more, living in the urban area of the municipality and registered in Brazil’s Family Health Strategy. Individuals were excluded based upon the following criteria: a score < 12 points on the Mini-Mental State Examination (MMSE) [24], using the adapted version for the Brazilian population; inability to ambulate, even with the assistance of the cane or walker; severe difficulty in visual and auditory acuity, according to the interviewer’s perception; wheelchair dependence and severe sequelae of cerebrovascular accident with localized loss of strength.

### Data collection procedures

The research team was composed of health professionals and academics from the Federal University of the Triangulo Mineiro and the State University of Bahia, Teixeira de Freitas campus. Researchers underwent extensive training on the study procedures before starting the data collection phase. Data collection for the present study occurred from July to October 2015. Community Health Agents of Alcobaça helped in identifying eligible individuals from the Family Health Strategy database. These individuals were later contacted by the ELSIA researchers. For contacting the potential participants, a door-to-door approach was used based on the contact information provided by the Community Health Agents. Potential participants were informed of the study objectives and procedures. Those interested in participating signed the written informed consent document, which was previously approved by the Ethics Committee in Human Research from the Federal University of Triangulo Mineiro.

Data collection consisted of a battery of physical performance tests (handgrip strength and gait velocity), anthropometric measures (weight, height) and an individual interview questionnaire on sociodemographic factors, regular physical activity level and sedentary behavior, as described next.

### Frailty

Frailty was diagnosed according to the adapted version of the original Cardiovascular Health Study model, considering the following four components [2]: 1) unintentional weight loss; 2) exhaustion evaluated by self-report of fatigue; 3) muscle weakness; 4) slowness assessed by slow walking speed.

Unintentional weight loss: Unintentional weight loss was assessed by the following question: “In the past year, have you lost more than 4.5 kg unintentionally (i.e., no diet or exercise)?” An answer of “yes” met the criterion for frailty in this category, adding one point to the overall assessment of frailty.

Exhaustion: Exhaustion was defined based on the following two questions from the Geriatric Depression Scale (Short Form), adapted for the Brazilian population: *“Did you stop doing many of your activities and interests?”* and *“Do you feel full of energy?”* [25]. A positive answer to the first question and/or a negative answer to the second question were considered to be signs of exhaustion/fatigue, and one point was added to the assessment of frailty.

Muscle weakness: Handgrip strength was assessed using a SAEHAN hydraulic dynamometer (Saehan Corporation SH5001, Korea). The test was performed according to the recommendations of the American Society of Hand Therapists. Briefly, the individual sat in a chair, with a back. The elbow was flexed at 90°, the forearm was in a neutral position, and the wrist was extended between 0 and 30°. The movable handle was in position II for women or position III for men. A verbal command was given by the examiner for the participant to begin the test. Participant then pressed the handle of the dynamometer with the highest force possible and held it for 6 s. Three measures in kilograms/force (kgf) were obtained for the dominant hand and the average of the three measures was reported. The cut-off points by Fried et al. [2], adjusted for sex and body mass index, were used to classify handgrip strength.

Slowness: Slowness was assessed by time in seconds to complete a 4.57-m walk test. Slowness was adjusted for sex and height. The following cut-off points were adopted: a time greater than or equal to 7 s for men of less than or equal to 173 cm; a time greater than or equal to 6 s for men taller than 173 cm; a time greater than or equal to 7 s for women with a height of less than or equal to 159 cm and a time greater than or equal to 6 s for women taller than 159 cm. Individuals who scored above the cut-off point in the walking test and those who were unable to perform the test due to physical limitations were considered to be positive for slowness, and one point was added to the overall assessment of frailty.

Frailty was scored through an ordinal variable system with scores ranging from 0 to 4 points. Scoring for each of the four frailty criteria was performed. The overall score was classified according to the following classification scheme: 0 to 2 points = not frail, and ≥ 3 points = frail [2]. Overall, 457 older adults provided data for frailty assessment and were included in the analysis.

### Physical activity and sedentary behavior

Physical activity and sedentary behavior were measured using the long form of the International Physical Activity Questionnaire, adapted for Brazilian older adults [26, 27]. The cut-off point of 150 min/week of moderate to vigorous intensity physical activity [28] was used to characterize physical activity level (≥150 min/week = sufficiently active and < 150 min/week = insufficiently active). For sedentary behavior, participants reported the time spent sitting on weekdays and weekend days [29]. A weighted average [(week × 5) + (weekend × 2)]/7 was used to estimate time spent in sedentary behavior during a typical day. The75th percentile (≥75th percentile) of sitting time [30], corresponding to 540 min/day in the present study, was used as the cut-off point for classification of excessive sedentary behavior.

### Sociodemographic variables

Sociodemographic variables consisted of age group (60 to 69, 70 to 79, 80 to 89, and 90 years or older), gender (male, female), marital status (single, married/living with partner, widower, divorced/separated), years of schooling (> 4 years, ≤4 years) and family arrangement (living alone, accompanied).

### Data analysis

Data were entered in duplicate in the Epidata software (version 3.1b). Statistical analyses were performed using the software Statistical Package for Social Sciences (SPSS, version 21). Descriptive statistics were used to calculate frequency (absolute and relative), mean and standard deviation for the study variables.

To assess the association of frailty with physical activity level combined with sedentary behavior, a Poisson multivariate regression was performed with estimates of prevalence ratios (PR) adjusted for sociodemographic variables. A significance level of 5 and 95% confidence intervals (CI) were used for denoting statistical significance.

## Results

Of the 743 older adults individuals enrolled in Brazil’s Family Health Strategy in Alcobaça, 54 individuals refused to participate in the survey, 58 were excluded because they did not meet the inclusion criteria (six wheelchair users; 10 bedridden; 19 with previous diagnosis of diseases that made it impossible to perform the interview, for example, blindness, hearing loss and Alzheimer’s Disease; 14 with a score < 12 in the MMSE, eight with communication difficulties and one alcoholic), 158 were not located and 16 did not provide information on all the study variables. Overall, data from 457 older adults were analyzed in this study.

The study population consisted of 285 women (62.4%) and 172 men (37.6%). A total of 8.8% of participants (*n* = 40) were classified as frail. Participant age ranged from 60 to 97 years, with a mean age of 70.25 years (SD = 8.25). The remaining characteristics of the study population, according to the frailty phenotype, are shown in Table 1. It can be observed that frailty was significantly more frequent (37.5%, *n* = 15) in participants aged 70 to 79 years. Moreover, regarding physical activity level combined with sedentary behavior, frailty was more frequent (50%, *n* = 20) in the insufficiently active and excessive sedentary behavior category (< 150 min/week and ≥ 540 min/day).

**Table 1:** Distribution of sociodemographic and behavioral variables, according to the frailty

Variable	N	Non Frail n (%)	Frail n (%)	χ^2^
Age groups (years)				
60 to 69	254	243 (58.3)	11 (27.5)	^b^22.06
70 to 79	135	120 (28.8)	15 (37.5)	*P* = 0.0001
80 to 89	58	48 (11.5)	11 (25)	
90 or more	10	6 (1.4)	4 (10)	
Sex				
Male	172	159 (38.1)	13 (32.5)	^a^0.49
Female	285	258 (61.9)	27 (67.5)	*P* = 0.48
Marital Status				
Single	41	39 (9.4)	2 (5)	^a^5.14
Married	214	200 (48)	14 (35)	*P* = 0.16
Widower	122	106 (25.4)	16 (40)	
Divorced	80	72 (17.3)	8 (20)	
Family arrangement				
Alone	74	67 (16.1)	7 (17.5)	^a^0.05
Accompanied	383	350 (83.9)	33 (82.5)	*P* = 0.81
Years of Study				
> 4 years	145	137 (32.9)	8 (20.5)	^b^2.53
≤ 4 years	310	279 (67.1)	31 (79.5)	*P* = 0.11
PAL (min/week) and SB (min/day)				
≥ 150 and < 540	141	131 (31.4)	10 (25)	^a^13.9
< 150 and < 540	92	86 (20.6)	6 (15)	*P* = 0.003
≥ 150 and ≥ 540	105	101 (24.2)	4 (10)	
< 150 and ≥ 540	119	99 (23.7)	20 (50)	

^a^Pearson’s chi-square; ^b^ Chi-square for linear trend; *PAL* physical activity level; *SB* sedentary behavior

Table 2 shows the association of the frailty phenotype and combined physical activity level and sedentary behavior, controlled for sociodemographic characteristics. There was a positive association observed between frailty and increasing age, with frailty being more prevalent in individuals aged 90 years or older (PR = 5.48) (95% CI = 1.31 to 22.95). Regarding physical activity level and sedentary behavior, there was an association between frailty and individuals in the insufficiently active and excessive sedentary behavior category (< 150 min/weekand 540 min/day).

**Table 2:** Association between frailty and the combination of physical activity level and sedentary behavior controlled for sociodemographic characteristics

Variable	Wald chi-square	PR	CI 95%	p
Age groups (years)				
60 to 69		1		
70 to 79	4.44	2.34	1.06 to 5.17	0.035
80 to 89	4.74	2.89	1.11 to 7.51	0.029
90 or more	5.42	5.48	1.31 to 22.95	0.020
Sex				
Male		1		
Female	0.35	1.24	0.60 to 2.58	0.55
Marital status				
Single		1		
Married	0.24	1.47	0.31 to 6.93	0.62
Widower	0.23	1.45	0.31to 7.78	0.63
Divorced	0.91	2.13	0.45 to 10.0	0.33
Family arrangement				
Alone		1		
Accompanied	0.33	0.76	0.30 to 1.92	0.62
Years of study				
> 4 years		1		
≤ 4 years	0.94	1.49	0.66 to 3.35	0.33
^a^PAL (min/week) and SB (min/day)				
≥ 150 and < 540		1		
< 150 and < 540	0.108	1.15	0.49 to 2.71	0.74
≥ 150 and ≥ 540	0.021	0.89	0.19 to 4.13	0.88
< 150 and ≥ 540	6.020	2.83	1.23 to 6.52	0.01

*PAL* physical activity level, *SB* Sedentary behavior; ^a^Adjusted for sociodemographic variables (age, gender, marital status, home arrangement and years of study)

## Discussion

This is the first epidemiological study conducted with Brazilian older adults to provide information on the association between frailty and the combination of physical activity level and sedentary behavior. The results indicate that frailty prevalence increases with low physical activity level combined with excessive time spent in the sitting position.

Frailty is a broad and dynamic concept related to the compromise of multiple systems, leading to greater vulnerability to adverse health factors, such as physical dependence, falls, medication consumption and hospitalization [31]. For the diagnosis of frailty, in general, markers of physiological and physical deficits are considered, and tests that assess phenotypic markers are typically selected for their practicality and low cost [2]. The aging process and longevity have a direct influence on the state of frailty [32], as frailty is more prevalent among older individuals [33].

Regarding behavioral lifestyle variables, the results indicate an association between frailty and insufficient activity level combined with excessive time spent in sedentary behavior. Although there have been previous studies that examined the relationship between either physical activity or sedentary behavior alone and frailty [7, 16], this is among the few studies that have investigated the association between frailty and the combination of physical activity level and sedentary behavior.

It is widely accepted that insufficient physical activity is a risk factor for several health conditions, such as cardiovascular diseases, obesity, cancer, mental disorders and all-cause mortality [34]. In general, the majority of the world population is not meeting the physical activity recommendations of a minimum of 150 min/week of moderate intensity physical activity or at least 75 min/week of vigorous intensity physical activity [28]. The number of persons not reaching the physical activity recommendations is of a global pandemic scale [11]. In older adults, insufficient physical activity level is even more concerning, since this problem is highly prevalent in this stratum of the world population, which is expanding rapidly. Thus, insufficient physical activity level constitutes a risk factor for the health of these individuals [35]. However, sedentary behavior also appears in the literature as an important health risk factor, and this behavior is related to several adverse health outcomes in older adults [6, 36]. There is still a lack of studies in the literature on the interrelation of physical activity with sedentary behavior and how their combination affects the health of older adults [37, 38].

The positive association observed in this study between frailty and the combination of insufficient physical activity level and excessive time spent in sedentary behavior can be explained, in part, by the series of deleterious effects caused by these behaviors. These two distinct behavioral aspects, when combined, may exacerbate the physiological alterations resulting from the aging process, itself, leading to a decline in total energy expenditure, maximal oxygen consumption and resting metabolic rate [39]. In addition, these behaviors lead to a caloric overload and the accumulation of central adipocytes, which, in turn, become metabolically active when filled with fat-generating inflammatory molecules, reducing the production of anti-inflammatory adipokines. This process may result in the development of chronic diseases, adverse health factors and, consequently, frailty in older adults [13].

In a cross-sectional study conducted by the National Health and Nutrition Examination Survey, a United States cohort with adult and older adults demonstrated that both sedentary behavior and moderate-to-vigorous intensity physical activity are associated with frailty [6]. The study results indicated that reducing sedentary behavior by one hour/day with a concomitant increase of one hour/day of moderate-to-vigorous intensity physical activity potentiates the reduction of the risk for frailty [6]. Additionally, an isotemporal substitution study showed that replacing 30 min of sedentary behavior with an equivalent amount of light-intensity physical activity were associated with a 14% decrease in the risk for frailty in older adults [40].

Thus, corroborating the existent literature, our results indicate significant health benefits for older adults who adopt a more active lifestyle. This includes the fulfillment of the minimum recommendations of physical activity and reductions in sedentary behavior engagement. These lifestyles changes are important for a healthier aging, allowing older adults to live longer and independently. Future intervention studies aiming at improving the quality of life of older adults should target the modification of both physical activity and sedentary behavior. It is also important to highlight that prospective studies are warranted to further and more accurately examine the dose-response relationship between frailty and the combination of physical activity level and sedentary behavior.

Some possible limitations were inherent to this study, such as the cross-sectional delineation that prevented progress in the cause and effect relationship between the variables. However, it is known that cross-sectional studies have a positive aspect over some other analytical study strategies, which is the high number of participants. Another limitation was the use of a self-reported questionnaire, which may have underestimated or overestimated some information as a result of the low levels of schooling and motivational aspects of participants.

To mitigate these possible limitations, researchers underwent extensive training to minimize motivational interferences and to standardize the instructions to participants during the interview process.

## Conclusion

The results of this study indicate that frailty is more prevalent among individuals who present with insufficient levels of physical activity and, at the same time, spend excessive time in sedentary behavior. Strategies to encourage physical activity, aiming to prevent frailty, in older adults should concomitantly focus on reducing the time spent in sedentary behavior.

## Data Availability

Data can be requested directly to the authors of this manuscript; however, access is subject to the approval of the Federal University of Triangulo Mineiro.

## Abbreviations

CI: Confidence interval
ELSIA: Longitudinal Study of the Elderly Health of Alcobaça
MMSE: Mini-Mental State Examination
PAL: Physical activity level
PR: Prevalence ratio
SB: Sedentary behavior
